# Size distributions of intracellular condensates reflect competition between coalescence and nucleation

**DOI:** 10.1038/s41567-022-01917-0

**Published:** 2023-02-02

**Authors:** Daniel S. W. Lee, Chang-Hyun Choi, David W. Sanders, Lien Beckers, Joshua A. Riback, Clifford P. Brangwynne, Ned S. Wingreen

**Affiliations:** 1 Lewis-Sigler Institute for Integrative Genomics, Princeton University, Princeton, NJ USA; 2 Department of Chemical and Biological Engineering, Princeton University, Princeton, NJ USA; 3 Howard Hughes Medical Institute, Princeton, NJ USA; 4 Department of Molecular Biology, Princeton University, Princeton, NJ USA; 5 Present Address: Department of Bioengineering, University of California, Berkeley, CA USA; 6 Present Address: Department of Molecular and Cellular Biology, Baylor College of Medicine, Houston, TX USA

**Keywords:** Biophysics, Self-assembly

## Abstract

Phase separation of biomolecules into condensates has emerged as a mechanism for intracellular organization and affects many intracellular processes, including reaction pathways through the clustering of enzymes and pathway intermediates. Precise and rapid spatiotemporal control of reactions by condensates requires tuning of their sizes. However, the physical processes that govern the distribution of condensate sizes remain unclear. Here we show that both native and synthetic condensates display an exponential size distribution, which is captured by Monte Carlo simulations of fast nucleation followed by coalescence. In contrast, pathological aggregates exhibit a power-law size distribution. These distinct behaviours reflect the relative importance of nucleation and coalescence kinetics. We demonstrate this by utilizing a combination of synthetic and native condensates to probe the underlying physical mechanisms determining condensate size. The appearance of exponential distributions for abrupt nucleation versus power-law distributions under continuous nucleation may reflect a general principle that determines condensate size distributions.

## Abstract (web summary)

Biomolecular condensates play a role in cellular processes and their size affects reaction pathways. The size distribution is connected to varying contributions of nucleation and coalescence.

## Main

Condensates of biological macromolecules play critical roles in many biological processes, including ribosome synthesis^1^, DNA organization^2^ and repair^3^ and stress responses^4,5^. In the context of metabolism, co-clustering enzymes into condensates can increase the efficiency of reactions by spatially co-localizing enzymes with their substrates^6^, but only for a restricted range of condensate sizes^7^. Similarly, large deviations from typical condensate sizes, which have been described for many nuclear condensates, including nucleoli^1^, Cajal bodies^8^ and nuclear speckles^9^, are associated with dysfunction and pathology. For instance, increased nucleolar size is associated with increased ribosome biogenesis^10^ and has been associated with both cancer^11^ and Hutchinson–Gilford progeria^12^. Moreover, condensates are strongly linked to pathological aggregation diseases such as Alzheimer’s and ALS. In these cases, there are many remaining questions about how large condensates might nucleate irreversible aggregates, and whether large aggregates are themselves pathological or merely end-stage outcomes from smaller pathological assemblies.

In non-living colloidal systems, the kinetics of coarsening processes are well known to be inherently linked with and to generally determine cluster size distributions^13–16^. By contrast, although biomolecular condensates have been observed in living cells for many years, their size distributions and coarsening dynamics have been rarely examined in detail. Among the few previously studied examples are the nucleoli in *Xenopus laevis* and in cultured human cells, which have been shown to have power-law size distributions^1,17^. Interestingly, such broad, scale-free size distributions are at odds with the suggestion that endogenous condensates have well-defined, functionally regulated sizes. However, in more typical rapidly dividing mammalian cells, nuclear bodies (such as nucleoli and speckles^8,18^) tend to appear following mitosis and coalesce^19^, but mechanical constraints probably due to chromatin result in subdiffusion and slow coarsening, limiting the growth of droplets on physiological timescales^20–23^. This picture is consistent with other recent work that has taken advantage of synthetic nuclear condensates, which form quickly^24^ but grow and coalesce slowly due to their constrained, subdiffusive motion^21^.

In some pathological contexts, cytoplasmic aggregates known as inclusion bodies or aggresomes are continuously produced, potentially providing a continually changing size distribution. For example, in the case of Huntington’s disease, the progressive misfolding of mutant Huntingtin protein exhibiting abnormally large stretches of polyglutamine or ‘polyQ’ repeats leads to the steady accumulation of material into irreversible aggregates^25^. Although they have more solid-like material properties than most physiological condensates, pathological aggregates can also generally move throughout the cell, and stick or fuse on collision, forming progressively larger structures that may even span the entire cell^26^ and are associated with neuronal dysfunction^27^ and cytotoxicity. How such steady accumulation of aggregating material is similar to or differs from the coarsening dynamics of physiological condensates, as well as the physical and biological principles that underlie such phenomena more generally, remains poorly understood.

Here we combine live-cell experiments and simulations to elucidate general principles linking condensate growth dynamics and size distributions. We demonstrate that endogenous nuclear speckles, which exhibit fast nucleation followed by gradual coalescence, yield an exponential size distribution, which can also be recapitulated in an engineered intracellular phase-separating system. By contrast, cytoplasmic Huntingtin aggregates reveal that continuous material production instead leads to a power-law distribution. We describe the differences that account for the scaling forms of the condensate size distribution in terms of a ‘preferential attachment’ effect, where merger probability and consequently growth rate are directly related to the condensate size. These findings may provide an insight into how cells biophysically regulate the size and number of condensates to generate a range of distributions, including exponentials and power laws.

## Results

### Nuclear condensates exemplify exponential size distributions

**Fig. 1:**
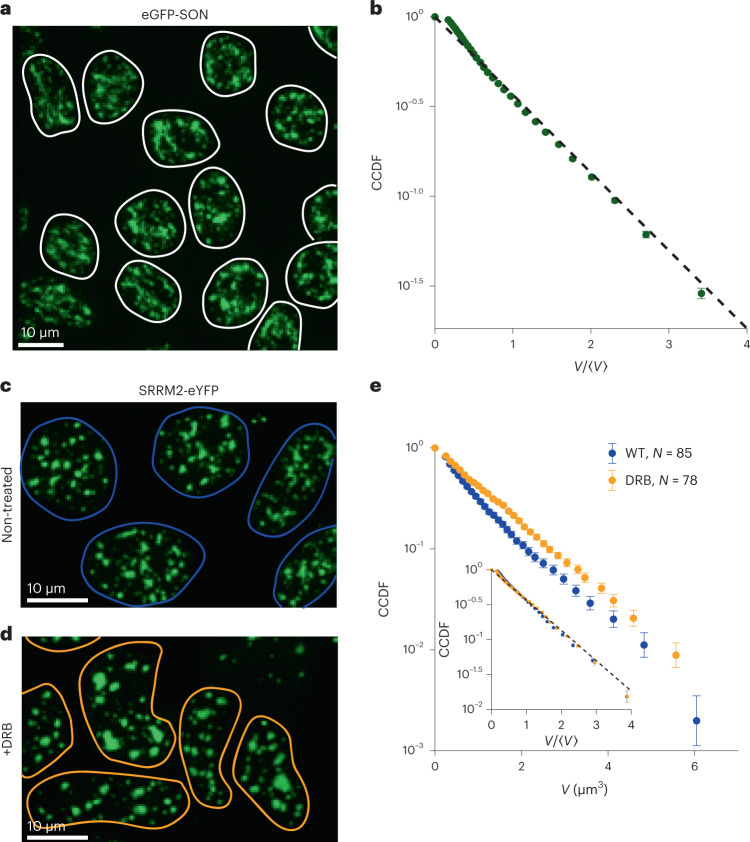
Endogenous nuclear speckles display exponential distributions before and after transcriptional inhibition. **a**, Exemplary maximum-projected image of iPSCs tagged with eGFP-SON and imaged in three dimensions. Nuclear outlines are highlighted in white. **b**, For each nucleus (*N* = 453), speckles were segmented in three dimensions, and the CCDF was calculated, rescaled by mean speckle volume in each nucleus and averaged over the nuclei, revealing good agreement with an exponential distribution (dashed line). The error bars reflect the standard error of the mean (s.e.m.) over the cells. **c**, Nuclear speckles were labelled in HEK-D cells by tagging SRRM2 with eYFP and imaged in three dimensions; nuclear outlines are highlighted in blue. **d**, Treatment with the transcriptional inhibitor DRB resulted in larger, brighter speckles and aberrant nuclear morphology. Nuclear outlines are highlighted in orange. **e**, CCDF was calculated for both non-treated control (*N* = 85; blue points) and following 4 h of treatment with 50 μg ml^–1^ DRB (*N* = 78; orange points), resulting in an increase in average speckle size from 0.92 ± 0.02 to 1.16 ± 0.03 µm^3^ (mean ± s.e.m.), giving linear CCDFs with slightly different slopes on a semi-log plot. Collapsing by rescaling by the average speckle volume (inset) confirms that both distributions remain exponential. The error bars reflect the s.e.m. over cells.

We first sought to determine the distribution of sizes of an endogenous nuclear condensate, namely, the nuclear speckle. Previous work suggested that speckles form quickly after mitosis and grow by coalescence^8^, thus acting as a model for the dynamics of a typical condensate. We imaged stem cells (induced pluripotent stem cells (iPSCs)) in which SON—a known marker of speckles^28^—was endogenously tagged with a green fluorescent protein (GFP) (Fig. 1a), since overexpressing condensate constituents can alter the condensate size^29^. We then segmented the speckles in three dimensions and quantified the probability density *f*(*V*, *t*) of condensate volume *V* at time *t*. Because the size distribution is highly sensitive to binning and cell-to-cell variation, we first considered the cumulative distribution function (CDF) of condensate sizes for each cell:

$$F\left( {V,t} \right) = \mathop {\smallint }\nolimits_0^V f\left( {V^\prime ,t} \right)\mathrm{d}V^\prime {{{\mathrm{,}}}}$$

and rescaled these distributions by each cell’s average condensate volume. We averaged over the rescaled distributions to account for cell-to-cell variation and finite sampling, and plotted the complementary cumulative distribution function (CCDF) as CCDF = 1 – *F*. We found that the nuclear speckle CCDF as a function of condensate volume *V* is linear on a semi-log plot for all timepoints, implying that these condensates are well described by an exponentially decaying size distribution (Fig. 1b, dashed line).

**Extended Data Fig. 1:**
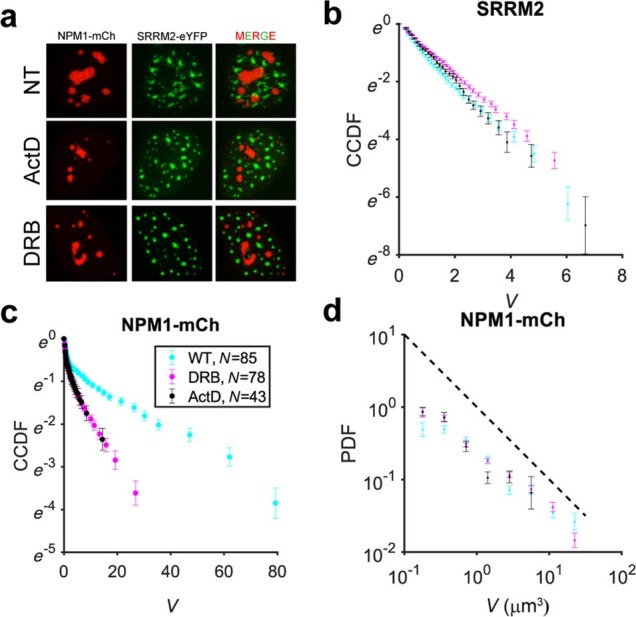
Analysis of endogenous nuclear body size distributions upon transcriptional inhibition. **a**, HEK-D cells were endogenously tagged with SRRM-2 to label nuclear speckles and were lentivirally induced to express exogenous NPM1-mCh to label nucleoli. Cells were treated with either 50 μg/mL DRB for 4 hours, or with 10 μg/mL actinomycin-D for 2 hours. Example nuclei are shown for each condition and channel. **b**, Nuclear speckles demonstrated exponential distributions as seen by plotting the CCDF for all three drug conditions; ActD decreased speckle size to $1.05 \pm 0.03\;{\upmu}m^3$ (mean ± s.e.m., $N = 43$ nuclei) compared to nontreated control ($\sim 1.16\;{\upmu}m^3$), but still larger than DRB treated cells $(\sim .92\;{\upmu}m^3)$. **c**, Unlike speckles, nucleoli strongly deviate from an exponential, exhibiting nonlinear behaviour in the semi-log CCDF. Nucleoli treated with transcriptional inhibitors were much smaller than the nontreated control, whose average volume was $11.6 \pm 0.8\;{\upmu}m^3$($N = 85$). The average volume after treatment with DRB was $4.0 \pm 0.2\;{\upmu}m^3$ ($N = 78$), and $0.97 \pm .02\;{\upmu}m^3$ after treatment with ActD ($N = 43$). **d**, For nucleoli, binning and averaging demonstrates that a power-law size distribution with a slope slightly above −1 (dashed reference line) is exhibited over a range of approximately 1-1.5 decades, consistent with literature^16^. As above, data is presented as mean (N = 85, 78 and 43 for WT, DRB and ActD respectively) and error bars report s.e.m.

To further interrogate this behaviour in a more tractable cell line, we utilized a monoclonal HEK293-derived (‘HEK-D’; Methods) cell line with eYFP-tagged SRRM2, another speckle marker (Fig. 1c)^26^, and expressing mCherry (mCh)-tagged NPM1 to mark the nucleoli. We first verified that an exponential was still observed in this cell line (Fig. 1e, dashed line). Previous work^9^ suggested that treatment with the transcriptional inhibitor 5,6-dichlorobenzimidazole 1-β-D-ribofuranoside (DRB) accelerates collisions between speckles, facilitating fusion. We asked whether this increase in dynamics would alter the form of the size distribution. Following 4 h of treatment with DRB, we find an increase in mean speckle size from 0.92 ± 0.02 µm^3^ (mean ± s.e.m. over 85 cells) in the non-treated control to 1.16 ± 0.03 µm^3^ (78 cells), consistent with the literature. However, despite this average size increase, both distributions were exponential (Fig. 1e). Similar results were observed using actinomycin D (Extended Data Fig. 1). By contrast, in nucleoli, we observed a power-law distribution, that is, *f*(*V*) ~ *V*^*k*^, with a slope *k* ≈ –1 (Extended Data Fig. 1)^17^, and a marked decrease in nucleolar size after both ActD and DRB treatment^30^, consistent with previous work^9,17^.

**Fig. 2:**
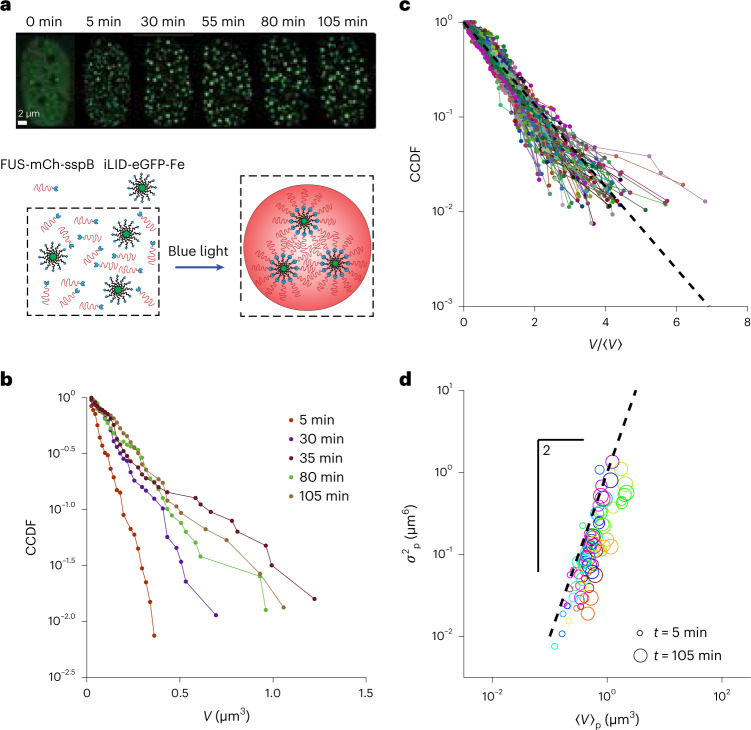
Synthetic nuclear condensates display exponential distributions as they coarsen. **a**, Synthetic Corelet system produces nuclear condensates on demand. The FUS_N_-mCherry-sspB and iLID-eGFP-ferritin fusion proteins are co-expressed in the nucleus. In the presence of blue light, sspB and iLID bind quickly, decorating the 24-mer ferritin core with oligomerized FUS_N_, driving oligomerization and phase separation (top row). **b**, CCDF of condensate volumes for the nucleus shown in **a** plotted on a semi-log scale reveals a progressively decreasing slope as the droplets coarsen in time and the mean condensate size increases following the initial quench (<5 min). **c**, For 18 nuclei, CCDFs rescaled by mean condensate volume in each nucleus were obtained at the timepoints depicted in **b** (reanalysed data from another work^21^) and compared with the expectation for an exponential distribution (dashed line). **d**, To further verify that the droplet size distributions are exponential, we calculated the mass-weighted or particle-wise mean and variance, defined as $\left\langle V \right\rangle _\mathrm{p} = \frac{{\Sigma p_VV^2}}{{\Sigma p_VV}}$ and $\sigma _\mathrm{p}^2 = \left\langle {\left\langle {V^2} \right\rangle _\mathrm{p}- \left\langle V \right\rangle _\mathrm{p}} \right\rangle$, respectively. These quantities were calculated for each nucleus every 25 min starting from 5 to 105 min following initial activation; the variance was plotted against the mean on a log–log plot and compared with the expected slope of 2 for an exponential distribution (dashed line).

**Extended Data Fig. 2:**
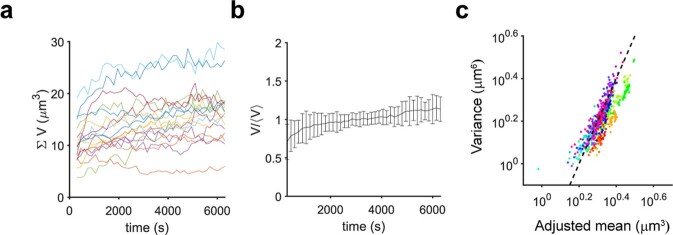
The Corelet system maintains a relatively constant amount of dense phase material and closely follows an exponential distribution. **a**, For 18 nuclei, the total volume of Corelet condensates in each nucleus was calculated and plotted over time (imaging every 3 seconds following the first 5 minutes of activation). **b**,To visualize overall changes in total condensate volume, in each of the 18 nuclei the total Corelet condensate volume was normalized by the time-averaged total condensate volume in that nucleus and then averaged over nuclei. Error bars reflect standard deviation. **c**, In the main text, it was noted that the mean in Fig. 2D is horizontally shifted from the expectation line; however, for an exponential distribution a shift in the mean can be attributed to a minimum experimentally detectable size, for example, due to the diffraction limit (see Supplementary Note). The mean condensate size was therefore adjusted by adding the minimum detectable volume, conservatively corresponding to a sphere of approximately $0.3\;{\upmu}m$ in radius, and plotted with respect to the variance on a log-log plot for comparison to the expectation from an exponential distribution (dashed line).

To examine the origin of these distributions in an even more experimentally tractable platform, we utilized the Corelet optogenetic system whose nucleation and coarsening dynamics have been well characterized^21,23,31^. Briefly, the Corelet system consists of two components (Fig. 2a): a 24-mer ferritin core whose monomers are fused to a GFP and an improved light-induced dimer (iLID) domain plus a protein, here the intrinsically disordered region of FUS, fused to an mCh fluorophore and a stringent starvation protein B (sspB) domain. On blue-light activation, the iLID and sspB heterodimerize, resulting in the oligomerization of FUS intrinsically disordered region, which drives phase separation within seconds after light activation^31^. Using this biomimetic system expressed in human osteosarcoma (U2OS) cells, we activated and imaged for 105 min (ref. ^21^); we observed that the CCDF is again consistent with an exponentially decaying size distribution. By examining the distribution as a function of time since light-initiated phase separation, we find that for a particular cell, the CCDF retains the linear shape on a semi-log plot, but changes the slope as the condensates coarsen and the average condensate size grows (Fig. 2b). To confirm that this behaviour was consistent across the cells and timepoints, we rescaled each CCDF by mean condensate size 〈*V*(*t*)〉 for each cell and time, revealing that these distributions obeyed the same scaling, all of them collapsing onto a line with a slope of −1 (dashed line) corresponding to an exponential distribution (Fig. 2c). Finally, to verify the Corelet exponential cluster size distribution, we also calculated and compared the volume-weighted or ‘particle-centric’ mean 〈*V*_p_〉 and variance $\sigma _\mathrm{p}^2$ for each nucleus and timepoint. These quantities are defined as the moments of the cluster size distribution weighted by the size of each droplet, that is, $\left\langle V \right\rangle _\mathrm{p} = \frac{{\Sigma V^2f}}{{\Sigma Vf}}$ and $\sigma _\mathrm{p}^2 = \left\langle {\left\langle {V^2} \right\rangle _\mathrm{p} - \left\langle V \right\rangle _\mathrm{p}} \right\rangle$, which should be less noisy but obey the same relation as the cluster-centric distribution for a system with fixed density clusters (for example, liquid–liquid phase separation). We note that the points fall along the line $\left\langle {V^2} \right\rangle _\mathrm{p} = \sigma _\mathrm{p}^2$ expected for an exponential distribution (Fig. 2d, with a slight horizontal shift that can be explained by the bias in the mean arising from a minimum detection size; Supplementary Note and Extended Data Fig. 2).

### Exponential distributions are independent of subdiffusion

**Fig. 3:**
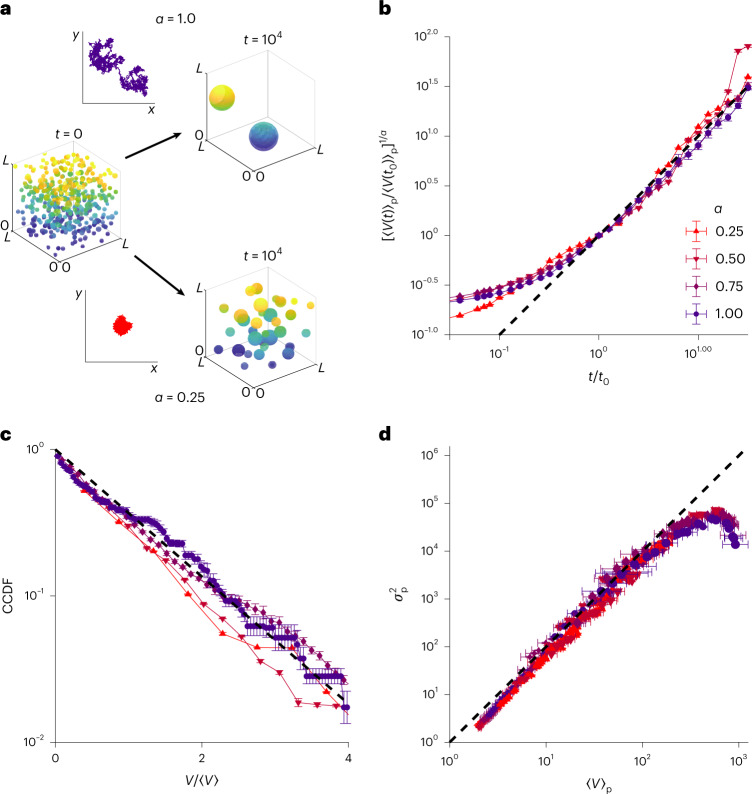
Monte Carlo simulations of subdiffusive coalescence also produce exponential distributions. **a**, Small spheres were randomly placed in a box with periodic boundary conditions and allowed to coalesce into larger spheres on collision. Sphere dynamics are dictated by fractal Brownian motion (example trajectories), resulting in faster coalescence for larger subdiffusive exponent *α* (Methods). **b**, Simulations of 1,000 spheres with initial volumes drawn from a uniform distribution between 1 and 2 were run for 10^5^ timesteps at a volume fraction of 5%, for varying subdiffusive exponents *α* from 0.25 to 1.00. To test the prediction that the mean $\left\langle {V(t)} \right\rangle _{{{\mathrm{p}}}}\approx \left\langle {V(t_0)} \right\rangle _{{{\mathrm{p}}}}\left( {\frac{t}{{t_0}}} \right)^\alpha$, we normalized the particle-centric mean by the particle-centric mean at *t*_0_ raised to 1/*α*, resulting in a collapse onto a line of slope 1 (dashed line). The error bars reflect the s.e.m. over 20 simulation replicates per condition. **c**, Distributions obtained as in **b** were averaged over 20 replicates before calculating the CDF and CCDF. The CCDF, rescaled by the mean sphere volume in each simulation condition, was plotted on a semi-log scale for comparing with an exponential (dashed line). **d**, Particle-wise mean and variance of sphere volume for each simulation condition, with different inputs Δ*r* (average diffusive step size, with input values of 0.1, 0.5 and 1.0), *α* (values of 0.25, 0.50, 0.75 and 1.00) and timepoints (from 10^2^ to 10^5^), plotted against each other (as in Fig. 2d), and compared with the expected slope of 2 for an exponential distribution (dashed line). The error bars reflect the s.e.m. over 20 simulation replicates per condition.

**Extended Data Fig. 3:**
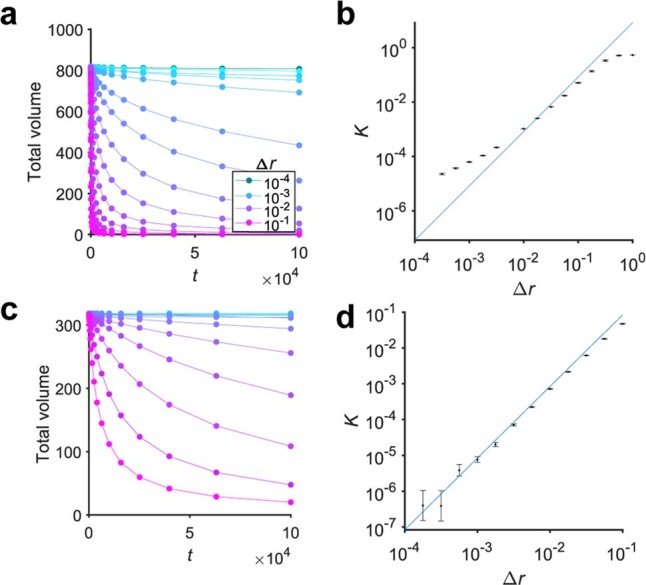
Control simulations validate merger rates in coalescence simulations. **a**, To compare the merger rate of spheres in our simulation framework to that predicted by analytical theory, we first modified the simulation such that spheres do not change in size upon merger, effectively lowering the amount of total material in the system over time but giving a system with a single value of *K* instead of a function that would vary with the size of the merging spheres, for ease of comparison to theory. We performed this simulation beginning with a system of 1000 spheres each of volume 1; spheres overlapping in the initial condition were replaced by a single sphere, and the system was allowed to evolve according to the specified diffusive step size. Total volume was plotted after the initial condition was resolved. **b**, The theoretically predicted merger rate (blue line; see Supplementary Note) was plotted for a range of diffusive step sizes and compared to the empirically calculated quantities (black points), finding agreement over a limited range of step-size values. Error bars reflect standard error of the mean over 20 replicates. **c**, To determine the source of disagreement between theoretical prediction and apparent merger rate in **b**, we first performed a “pre-run” of 2×10^4^ timesteps at a step size $\Delta r = 0.03$ starting with an initial condition identical to that in **a** for all conditions before collecting data, effectively “diluting” the system by nearly 5-fold. **d**, The collision rate constant was calculated for a range of step-size values following the pre-run protocol. Data represent mean and error bars are s.e.m. We found that this pre-run protocol gave a closer agreement with the theoretical prediction over a larger range of values, suggesting that the disagreement seen in **b** is merely due to the initial condition having fewer than predicted mergers, rather than the implementation of the dynamics themselves.

To quantitatively understand the underlying basis for the exponential size distribution of the condensates described above, we developed Monte Carlo simulations to replicate and manipulate the coagulation dynamics. We hypothesized that, in general, a fast quench—where many small condensates nucleate nearly simultaneously, followed by growth via slow coagulation—would produce an exponential distribution. In our simulations, we first generated *N* (generally, 1,500) spheres whose initial volumes *v*_0_ were sampled from a uniform distribution in the range of [1, 2], which were randomly placed in a cubic box of side length *L*, such that the input volume fraction is *v*_f_ = *Nv*_0_/*L*^3^ (generally set to 0.05). Spheres whose initial positions overlapped were merged by replacing them with a larger sphere centred at their centre of mass and conserving volume (Fig. 3a). The spheres were then allowed to move following the fractional Brownian motion trajectories generated by wavelet synthesis^32^ with an input exponent *α*, that is, $\left\langle{\boldsymbol {r}}^{2}\right\rangle = {{2dD}{\tau}^{\alpha}}$ for displacement ${\boldsymbol {r}}$, dimension *d*, diffusion coefficient *D* and time lag *τ*. Thus, the simulations could be tuned to mimic subdiffusive (*α* < 1) or diffusive (*α* = 1) behaviour, a physiologically relevant parameter, considering the ubiquity of subdiffusion in cells^21,33,34^. In the simulations, spheres diffused according to the specified dynamics and were merged on contact, consistent with the observation in the Corelet system that collisions nearly always result in coalescence. We found that the size-dependent rate of mergers agreed with predictions from coagulation theory (Extended Data Fig. 3)^35^.

We ran replicates over a range of (sub)diffusive conditions and calculated the distribution of volumes after a fixed time. First, we verified that the coarsening agreed with previous theory^21^, which predicted that *V*/*V*_0_ ≈ (*t*/*t*_0_)^*α*^; using the initial value of *t*_0_ = 100, the volume trajectories fell along a line with slope 1 for 1.0–1.5 decades in time (Fig. 3b). Then, to analyse the size distributions, we plotted the sphere size distribution and rescaled by the average sphere volume as in the analysis of the experimental data, observing exponential distributions under all the conditions for example timepoints (Fig. 3c). The particle-wise mean versus variance for all the timepoints beyond *t*_0_ was also plotted (Fig. 3d) for several values of diffusive exponent *α*, also yielding a line with a slope characteristic of an exponential distribution, with the exception of late times, when the number of spheres became very small. Thus, in simulations with a wide range of diffusive exponents and in experiments with various synthetic and endogenous condensates, we observed exponential distributions.

### Slow polyQ nucleation results in a power-law distribution

**Fig. 4:**
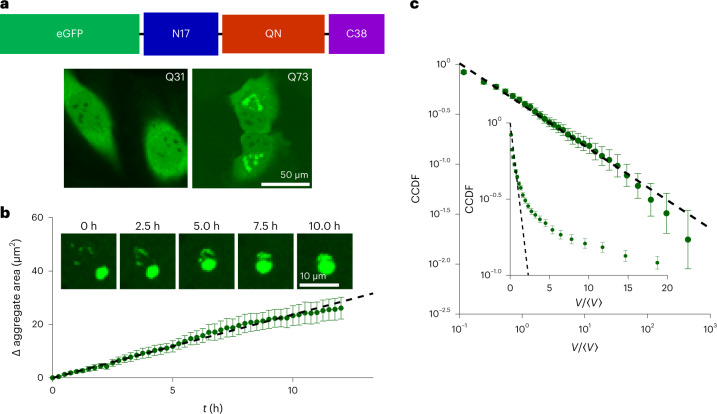
PolyQ aggregates exhibit slow-nucleation dynamics and a power-law cluster size distribution. **a**, HeLa cells were transduced with lentivirus to express eGFP-Htt-polyQ constructs of mutant Huntingtin exon 1 composed of N17, QN and C38 domains. Four days following transduction, cells infected with eGFP-Htt-Q31 had a diffuse GFP signal, whereas bright micrometre-scale puncta were evident in those infected with eGFP-Htt-Q73. **b**, Cells expressing eGFP-Htt-Q73 were imaged for 12 h. Aggregates were observed to merge on contact (inset). PolyQ growth was quantified over *N* = 5 cells imaged every 15 min. Subtracting the initial projected aggregate area and averaging revealed an increase in aggregate area over time at a per-cell rate of 0.04 ± 0.01 µm^2^ s^–1^ (error is expressed as a 95% confidence interval for a linear fit; *p* < 10^–^^52^ for an *F* test for comparing with a constant model). **c**, PolyQ aggregate cluster size distribution was calculated by averaging per-cell normalized cumulative distribution functions over cells (*N* = 114) between 4 and 6 days after lentiviral infection. The CCDF was fit to a power law, yielding CCDF(*V*) ≈ *V*^–0.41±0.02^ (error is expressed as a 95% confidence interval for linear fit; *p* < 10^–2^^5^ for an *F* test for comparing with a constant model). The CCDF was also compared with the expectation for an exponential distribution (inset).

**Extended Data Fig. 4:**
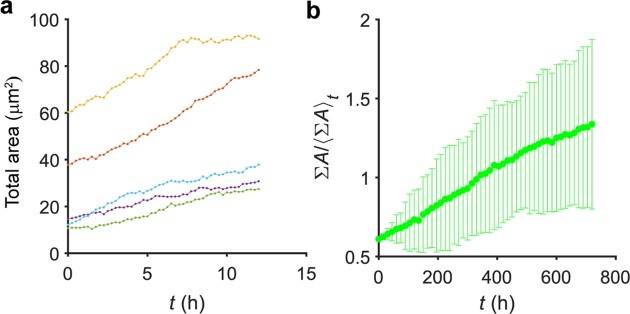
PolyQ dynamics reveal a large increase in aggregate material over time. **a**, Total PolyQ protein aggresome area was plotted over time for each of five cells, imaging every 15 minutes for 12 hours. **b**, To assess the overall change in aggregate material, the aggregate area normalized by the time averaged aggregate area in each cell was averaged over cells and plotted; error bars indicate standard deviation. The amount of aggregate material increases on average about twofold from the beginning to the end of the time course.

Our findings thus far suggest that the rapid nucleation of small condensates followed by slower coarsening due to coalescence generically results in an exponential distribution. To further elucidate how the relative timescales of nucleation and coagulation affect the scaling of the condensate size distribution, we next considered a contrasting system characterized by the continuous, sustained nucleation of new small condensates. Specifically, we utilized the Huntingtin polyQ exon 1 (Htt-polyQ) system, which—unlike the above systems—exhibits a slow and steady increase in the total aggregate material with time. Exogenous eGFP-Htt-polyQ protein was lentivirally expressed in HeLa cells to induce aggregate formation. Consistent with previous work^25^, eGFP-Htt-Q31 did not form aggregates, but eGFP-Htt-Q73 formed large aggregates in perinuclear regions of the cytoplasm within four days following infection (Fig. 4a). These aggregates were observed to nucleate over the span of hours and merge on contact (Fig. 4b). To quantify the production dynamics, we measured the total projected area of aggregated material as a function of time, finding a roughly constant average production rate of approximately 0.04 ± 0.01 µm^2^ s^–1^ (Fig. 4b), which corresponds to an increase of 2.28 ± 0.24 times over the observed interval (Extended Data Fig. 4); this contrasts with the Corelet system, which generally remained stable in the total condensate volume, following an initial nucleation period (Extended Data Fig. 2a,b).

**Extended Data Fig. 5:**
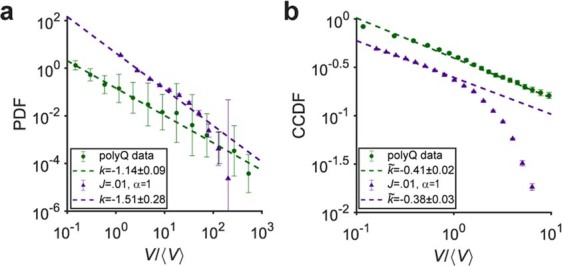
PolyQ size distributions compared to slow injection simulations. **a**, Probability distribution functions for both the polyQ experimental data and the slow injection simulation ($J = 0.01,\;\alpha = 1$) were calculated over 114 cells or 20 simulation replicates, respectively, and rescaled by the per-cell or per-simulation mean condensate size before averaging. The data was plotted (error bars are standard error of the mean) and fitted to power-law models with $f\left( V \right)\sim V^k$ (dashed lines, uncertainty is reported as 95% confidence interval of the linear fit). **b**, The CCDF was calculated for both simulation and experimental data sets once again, plotted (error bars reflect standard error of the mean) and fit to a power-law model such that ${{{\mathrm{CCDF}}}}\sim V^{\tilde k}$. Error is reported as 95% confidence interval of the fit. In the case of the simulation data, the fit was performed over the linear regime of the CCDF, from $10^{ - 1} < \frac{V}{V} < 10^0$, since the CCDF fell off due to finite size effects.

To examine the effect of these contrasting kinetics, we quantified the size distributions of polyQ aggregates. To calculate the per-cell distribution and avoid binning artifacts, a CDF was calculated for each cell and averaged over the cells. The resulting CCDF was well fit by a power law, that is, ${{{\mathrm{CCDF}}}} \approx V^{\tilde k}$ with exponent $\tilde k$ = –0.41 ± 0.02, noting that $k = \tilde k + 1$ thus corresponds to an exponent of −1.41 for the probability distribution function (Fig. 4c and Extended Data Fig. 5); the CCDF was poorly fit by an exponential (Fig. 4c, inset).

### Injection–collision rate ratio controls size distributions

**Fig. 5:**
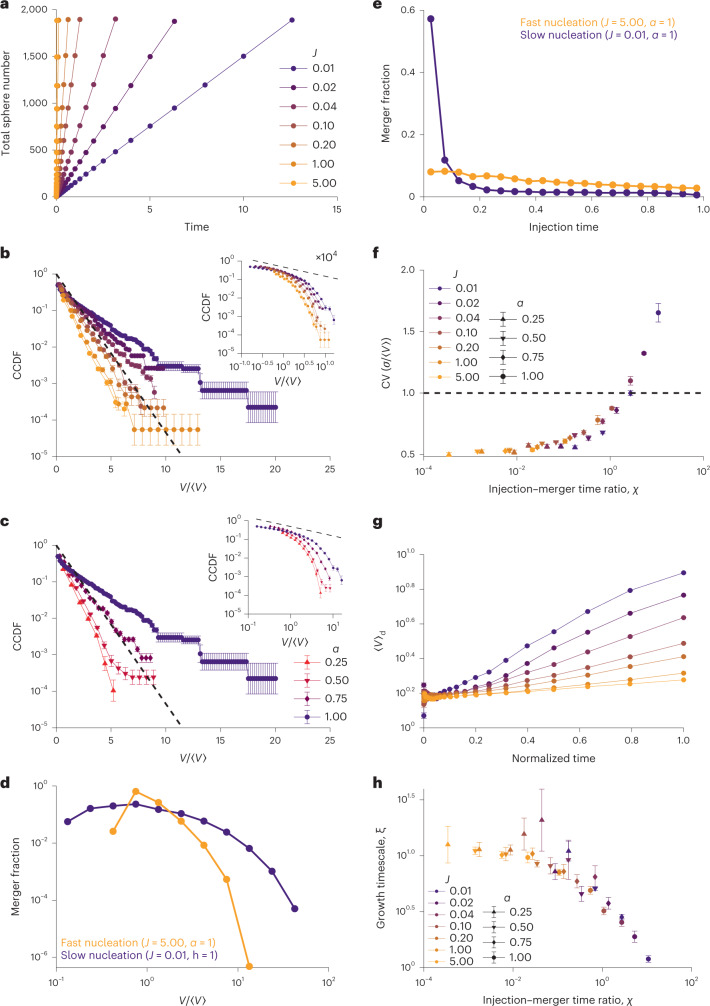
Simulations incorporating gradual material production demonstrate a power law. **a**, Simulations steadily introduced material at a rate of *J* spheres per unit time, starting with an empty cell, until 1,200 spheres were injected and a volume fraction of 4% was obtained. **b**, Cluster size distribution was calculated after the steady injection of 1,200 spheres and plotted (inset). Rapid injection produced an exponential distribution of sphere volumes, whereas slow injection gave a better fit to a power law with a slope of almost −0.5 based on the log–log plot of the CCDF (inset, dashed line). The error bars reflect the s.e.m. over 20 simulation replicates for each *J*. **c**, Injection simulations were run with *J* = 0.01 for varying values of diffusive exponent *α*. Plotting the CCDF demonstrates that for low *α*, an exponential distribution is obtained. The error bars reflect the s.e.m. over 20 replicates for each *α*. **d**, Mergers involving ‘monomeric’ spheres were analysed for *J* = 5.00 and *J* = 0.01 for *α* = 1. The distribution of sizes of spheres with which monomeric spheres merged and normalized by the mean sphere size at the moment of merger was calculated. The error bars reflect the s.e.m. over 960 simulation replicates per condition. **e**, Time of injection, normalized by simulation duration, for spheres with which monomeric spheres merged was calculated for 960 replicates. The injection time of a sphere is taken to be that of the earliest-injected sphere in its merger history. The error bars reflect the s.e.m. over 20 simulation replicates per *J* value. **f**, Coefficient of variation was calculated for each *α* and *J* and plotted against *χ*, the ratio of the injection to the merger timescale. The error bars reflect the s.e.m. **g**, Average sphere size was plotted as a function of time, normalized by the simulation duration for *α* = 1. The error bars reflect the s.e.m. (mostly smaller than the data points). **h**, For each condition, the growth timescale was calculated by linearly fitting the log of the average sphere size against normalized time to give *ξ*. Each *ξ* was compared with *χ*, the ratio of injection to merger timescales, for each *α* and *J*. The vertical error bars represent 95% confidence interval of the linear fits for 20 replicates per condition.

We hypothesized that the broad power-law distribution observed in the polyQ system is due to the similarity of the timescales for the appearance of new aggregates and for mergers of existing aggregates. To test this idea, we returned to our Monte Carlo simulations to model the slow nucleation of the polyQ system. Specifically, we modified the simulations such that spheres appear at a fixed injection rate of *J* spheres per timestep, rather than all at once at *t* = 0 (Fig. 5a). For a range of *J* values, we ran simulations until a fixed number of spheres were injected (the same for every simulation), and then calculated the CCDFs (Fig. 5b). Although the simulations varied in total duration as 1/*J*, we reasoned that the relevant timescale in the system was set by the diffusion of spheres; therefore, changing the total duration and injection rate effectively varied the relative rates of merger and material production. For relatively high values of *J* (*J* > 0.1), the resulting distributions were approximately exponential, but for low values of *J* (*J* < 0.1), the distributions were more power-law like (Fig. 5b), consistent with previous work^1,36^. In particular, we note that the *J* = 0.01 case produces a power-law-like probability distribution function with an exponent of *k* = –1.51 ± 0.28 (Extended Data Fig. 5), very close to the power-law exponent observed in our polyQ experiments. This suggests that the slow addition of new material drives the system towards a broader distribution of sizes, associated with a power-law distribution with exponent of *k* = –1.5. Similarly, we reasoned that if decreasing *J* results in a broader distribution because injection becomes slower relative to the merger, then conversely, slowing down the merger rate should similarly recover an exponential distribution. Therefore, we held *J* fixed at a value of 0.01 and varied the subdiffusive exponent in our simulations. We found that decreasing the subdiffusive exponent and thus slowing down mergers indeed yielded an exponential distribution (Fig. 5c).

Given that simply changing the rate of injection of new material was sufficient to change the form of distribution from an exponential to a power law, this provided an opportunity to track down the underlying mechanism responsible for these qualitatively different outcomes *in silico*. We hypothesized that in the case of low *J*, the first spheres to be injected have more opportunities to merge and consume smaller spheres, which might result in preferential attachment onto larger spheres—such preferential processes have been shown in a wide variety of contexts to generate power-law distributions^37–39^. We, therefore, analysed the collision events of the respective systems, selecting the extreme *J* = 5.00 and *J* = 0.01 cases. First, we specifically considered mergers involving ‘monomeric’ spheres, that is, spheres that had been injected and not yet undergone any mergers. We recorded the size of the spheres with which they merged, normalized by the mean sphere size at the moment of merger, finding that for *J* = 5.00, monomeric spheres typically merged with spheres close to the mean sphere size, whereas for *J* = 0.01, the distribution of merger partners was much wider, reflecting the bias of collisions towards spheres much larger than the mean (Fig. 5d). Furthermore, we calculated the time of injection for the spheres with which monomer spheres collided, normalized by the total duration of the simulation, finding that the merger fraction decreases linearly with injection in the fast-injection case (Fig. 5e). However, in the slow-injection case, the merger fraction was even more strongly dependent on the injection time of the merger partner, with approximately 80% of monomeric spheres merging with a sphere that had originally been injected within the first 25% of the simulation (Fig. 5e), matching our intuition that mergers were heavily biased towards ‘older’ spheres.

**Extended Data Fig. 6:**
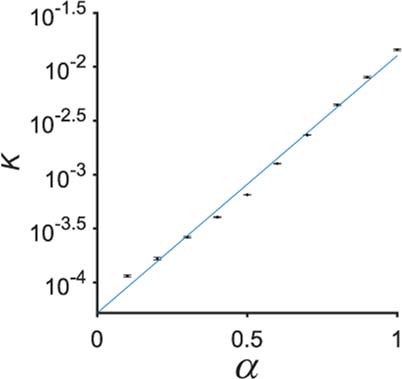
Empirical calculation of collision rate constant dependence on subdiffusive coefficient. The dependence of the collision rate constant *K* on the diffusive exponent *α* was empirically calculated by running 20 replicates of simulations each initially containing 1000 spheres, which did not change size upon merger (that is, identical to Supplementary Fig. S2a), with step size $\Delta r = .05$ and varying *α* from 0.1 to 1. *K* was calculated for each condition by taking the merger probability weighted by the sphere number density. Error bar reflects standard error of the mean over replicates. *K* was well fit by a linear model $(R^2 = 0.993)$ in semi-log, that is, ${{{\mathrm{log}}}}\left( K \right)\sim \alpha$ (blue line), which relation was used to calculate the ratio *χ* of injection to merger timescales (Fig. 5).

Based on this analysis, we hypothesized that the preferential attachment effect in this system was driven by the competition between mergers and injection. To generalize this to the wide, multiparametric space in this system, we derived an expression for a dimensionless number, namely, $\chi = \frac{{\tau _{{{{\mathrm{inject}}}}}}}{{\tau _{{{{\mathrm{merge}}}}}}}$, which compares merger and injection timescales predicted with the input parameters (Supplementary Note); for *χ* > 1, injection is slow and there is a strong bias towards collision with the largest spheres, whereas if *χ* < 1, merger is slow and collision probabilities are roughly size independent, resulting in an exponential size distribution. The merger timescale *τ*_merge_ is given by the time required to close the distance between the spheres by (sub)diffusion, that is, $\tau _{{{{\mathrm{merge}}}}} = \frac{1}{{K\left( \alpha \right)\rho }}$, where *ρ* is total number of injected spheres per system volume and *K*(*α*) is the collision rate constant, whose dependence on the diffusive exponent *α* is non-trivial and was empirically calculated via simulation (Extended Data Fig. 6). The injection timescale *τ*_inject_ is the time required to inject all the spheres into the system, that is, $\tau _{{{{\mathrm{inject}}}}} = \frac{\rho }{j}$, where $j = \frac{J}{{V_{{{{\mathrm{sys}}}}}}}$ is the rate of injection of new spheres per unit volume, and therefore,

$$\chi = \frac{{\tau _{{{{\mathrm{inject}}}}}}}{{\tau _{{{{\mathrm{merge}}}}}}} = \frac{{\rho ^2K\left( \alpha \right)}}{j}.$$

For each simulation condition, to characterize the distribution of sphere volumes at the time when the last new sphere was injected, we calculated the ratio of standard deviation to mean (also known as the coefficient of variation (CV)). For an exponential distribution, CV = 1, whereas a power-law-like distribution will have a larger CV. We then plotted CV against *χ* for each simulation condition (Fig. 5f) and found that all the conditions collapsed onto a single curve, matching our intuition that the ratio of timescales captured by *χ* governs the width of the final distribution of sphere volumes; we note that for very small values of *χ*, the CV is smaller because very few mergers occur and the distribution is restricted to almost entirely monomeric spheres.

We similarly anticipated that *χ* would control the evolution of the mean of sphere volume distribution in addition to its polydispersity. Returning to the steady-injection simulations, we found that the mean volume increased close to exponentially with time (Fig. 5g); we, therefore, defined a characteristic timescale *ξ* for the growth of the mean by fitting the semi-log plot of mean sphere volume to a line. We then compared *ξ* with *χ* for the full set of parameters and found that *ξ* is indeed dependent on *χ*, with *ξ* decreasing as *χ* increases (Fig. 5h).

**Extended Data Fig. 7:**
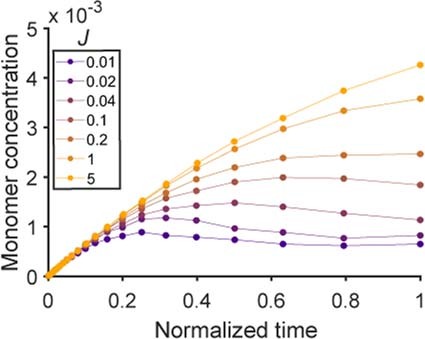
Monomer concentration reflects the preferential attachment effect. Monomeric sphere concentrations (that is, number of spheres which had been injected and not yet merged with any other sphere) were calculated for each injection rate *J* with $\alpha = 1$. For slow injection rates, the monomer concentration decreased at long times, while it continued to increase for fast injection rates as monomers accumulated more quickly than merger events occurred. For intermediate injection rates, where $\chi \sim 1$, the monomer concentration approximately reached a plateau.

Taken together, we conclude from this theoretical analysis that for *χ* < 1, that is, when the timescale of a typical merger is greater than the time required to inject all the material into the system, the distribution remains narrow and close to an exponential since mergers are sparse and occur between random pairs. Moreover, the growth of the mean is slow on the timescale of injection because the injected monomers accumulate with time, accounting for an increasingly large fraction of the population (Extended Data Fig. 7). By contrast, for *χ* > 1, many more merger events occur during the injection process, giving older spheres more merger opportunities, mimicking the polyQ case, resulting in a broader, more power-law-like distribution. In this case, the mean grows quickly on the timescale of injection as monomers are efficiently consumed by larger spheres (Extended Data Fig. 7).

### Preferential attachment generates power-law distributions

We next sought to investigate other mechanisms by which a preferential attachment effect might come into play. We considered that this effect can fundamentally be understood in the context of the simplest coagulation framework first analytically solved in another work^40^, where the relative rate at which spheres of particular volumes (*V*_1_, *V*_2_) with diffusion coefficients (*D*_1_, *D*_2_) collide can be described using a matrix known as a coagulation kernel *K*(*V*_1_, *V*_2_). For the diffusive spheres in our simulations, this is given by

$$K\left( {V_1,V_2} \right) = 4\uppi \left( {D_1 + D_2} \right)\left( {R_1 + R_2} \right),$$

noting that $V_i = \frac{4}{3}\uppi R_i^3$ and that in the typical Stokes–Einstein case, *D*_*i*_ ≈ 1/*r*_*i*_. We reasoned that in the fast-quench-and-coalescence case, all the simultaneously nucleating spheres were about the same size; for *R*_1_ = *R*_2_, *K* is constant as a function of sphere volume or radius and therefore mergers were ‘equal opportunity’, giving an exponential size distribution. However, in the slow-injection case, because the older spheres were much larger than the newly injected spheres, *R*_1_ ≫ *R*_2_ and therefore *K* ≈ *R*_1_/*R*_2_, meaning that small spheres tended to merge with large spheres, giving rise to a power-law distribution as observed in the slow-injection case.

We, therefore, sought to test whether artificially strengthening the collision bias towards larger spheres by directly manipulating the coagulation kernel could give rise to a power-law size distribution starting from the same narrow initial size distribution as in our previous fast-quench simulations, which produced exponential distributions. Indeed, the coagulation kernel is expected to vary with the conditions. It has been previously shown that in the case of the diffusion of individual monomers in a Rouse polymer^41^, or, experimentally, in the case of condensates embedded in chromatin^21^, *D*_*i*_ ≈ *R*^–0.5^, instead of *D*_*i*_ ≈ *R*^–1.0^ as prescribed by the Stokes–Einstein relation. It has furthermore been shown that in active suspensions with non-thermal forces that probably play a role in biological processes, the fluctuation–dissipation relation may not be obeyed, and thus, the Stokes–Einstein relation can be broken^42^.

**Fig. 6:**
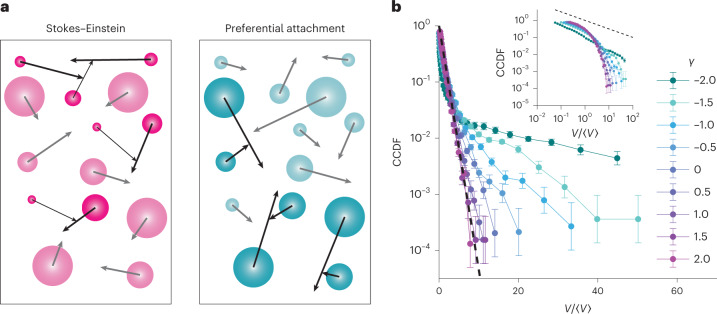
Preferential attachment effect results in power-law-like size distributions. **a**, Schematic depicting the preferential attachment effect. For the Stokes–Einstein diffusion of condensates, an increased radius of large condensates is compensated by a decrease in their mobility. Artificially inverting this relation results in increased collisions involving larger condensates, yielding a strong preferential attachment effect. **b**, Cluster size distributions were calculated varying the scaling exponent *γ* of the diffusion coefficient with sphere size (that is, $D\approx \frac{1}{{R^\gamma }}$) with *γ* ranging from −2 to 2 and were plotted as CCDFs in the semi-log scale to compare with an exponential (dashed line) and in the log–log scale to compare with a power law (inset). The error bars reflect the s.e.m. over 20 simulation replicates per condition.

Thus, we performed simulations for ordinary diffusion *α* = 1, but for each simulation, we set $D = \frac{{D_0}}{{R^\gamma }}$ for different values of *γ*, where *γ* = 1 corresponds to the standard Stokes–Einstein dependence of the diffusion coefficient on radius. For values of *γ* < 1, merger events become biased towards larger spheres since diffusion coefficients become less dependent on size, whereas larger spheres have larger collision radii. For *γ* < 0, spheres actually accelerate as they grow, further strengthening this bias (Fig. 6a).

**Extended Data Fig. 8:**
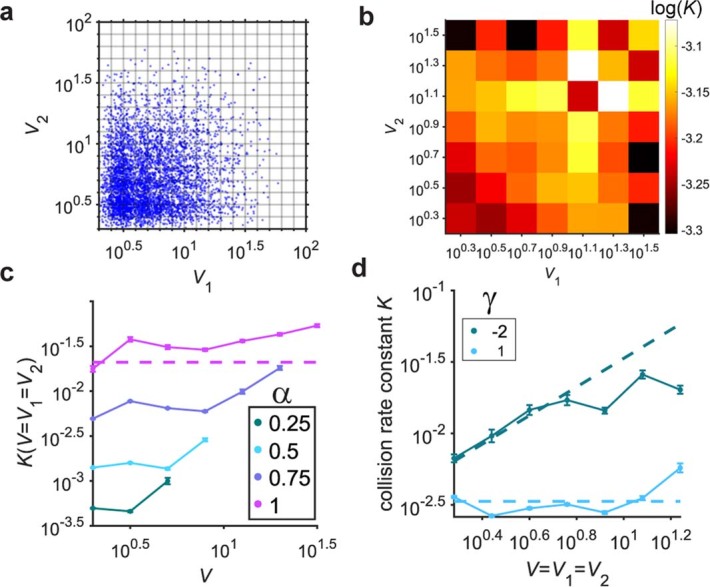
Calculating the coagulation kernel allows for quantification of the preferential attachment effect. **a**, To characterize the simulated merger dynamics, we first plotted the incidence of individual merger events between spheres of particular volumes for an example system (5000 randomly chosen events were plotted for visual clarity). **b**, To describe the apparent strength of the preferential attachment effect, we weighted each merger event by the number density of the sphere sizes involved in the merger, and averaged over each possible pair of sizes, giving an estimate of the collision rate constant or coagulation kernel *K*. **c**, The coagulation kernel diagonal was computed for 960 replicates of the fast-quench-and-coalescence simulation with 1000 spheres and plotted against theoretical expectation for ordinary diffusion (magenta dashed line), demonstrating that for all subdiffusive conditions, there is no strong preferential attachment effect, as expected. **d**, The diagonal of the kernel was calculated and plotted for simulations with $\gamma = 1$ and $\gamma = - 2$ for 1000 replicates. Both conditions were compared to and agreed well with theoretical expectation, that is, $K\left( V \right) = 16\pi {{\Delta }}r^2V^{\frac{{1 - \gamma }}{3}}$, where Δ*r* is the diffusive step size (dashed lines). Error bars are s.e.m.

We observed exponential distributions for *γ* > 1, consistent with our previous simulations, but as *γ* decreased below 1, we found progressively more power-law-like distributions (Fig. 6b). We then empirically calculated the coagulation kernel, estimating a collision rate constant by weighting each collision event by the inverse of the number density of spheres of that size. To quantify the dependence of merger probabilities on *γ*, we plotted the kernel diagonal against sphere size. For *γ* = 1, *K* was constant, consistent with analytical theory, but for *γ* = –2, we found that the diagonal increases with sphere size (Extended Data Fig. 8), suggesting that the merger probability increases with sphere size, resulting in preferential attachment. We also tested several subdiffusive exponents *α* and keeping *γ* = 1. In these instances, we found that the kernel diagonal remained constant as a function of radius for different values of *α*. This indicates that the distribution would be exponential regardless of the subdiffusive exponent. Indeed, this expectation is consistent with our simulations and our experimental observations in the Corelet system, where we observed exponential distributions for multiple values of *α*.

These results demonstrate the control of sphere size distribution by simply biasing the size dependence of the collision rates, independent of the rate of adding spheres to the system. We conclude that the scaling form of the size distribution generally reflects the strength of preferential attachment effect in growth kinetics, due to either material addition rate or size-dependent diffusion. Thus, quantifying the scaling form of condensate size distributions in live-cell experiments is sufficient to characterize the strength of the preferential attachment effect.

## Discussion

In this work, we elucidated the general principles underlying the size distributions of intracellular condensates, including those involved in vital biological processes. We described two drastically different, experimentally motivated mechanisms of condensate growth: a quench-then-coalesce model and a slow-nucleation model. We show that the size distributions of both can be accounted for by comparing the timescales of nucleation and coalescence.

Colloidal cluster size kinetics and cluster size distributions have previously been comprehensively studied^13,14,43,44^, leading to the description of two universality classes, identified as diffusion-limited cluster aggregation (DLA) or reaction-limited cluster aggregation (RLA). The limiting cases we identify here bear some similarity to DLA and RLA: DLA describes the power-law growth of the mean cluster size^13^ and a decaying size distribution^14^, the latter of which we observe to be an exponential in our quench-then-coalesce mechanism. By contrast, in the slow-nucleation/slow-injection case^14^, we observe an approximately exponential growth of the mean^13^ and a power-law size distribution as in RLA. However, unlike in DLA and RLA, material in the cell is constantly produced and exists in a viscoelastic medium, which are not considerations in colloidal systems. Nevertheless, we have demonstrated that these biologically motivated mechanisms can lead to outcomes quantitatively analogous to DLA and RLA.

Indeed, based on our results, we can quantitatively describe the evolution of condensates that quench and then coalesce as consistent with dynamic scaling, which was first associated with DLA by other studies^43,45^. This mechanism of growth generically yields an exponential distribution, independent of subdiffusive exponent or time, which implies that a single parameter—the average condensate volume 〈*V*〉—defines the distribution. The average condensate volume has previously been shown to grow in time as 〈*V*(*t*)〉 ≈ *V*_0_(*t*/*t*_0_)^*α*^ determined by the subdiffusive exponent *α*, following quench and nucleation to an average initial condensate volume *V*_0_ at some time *t*_0_ (ref. ^14^). We can, therefore, describe the distribution for the quench-then-coalesce mechanism simply as

$$f\left( {V,t} \right) = \frac{{t_0}}{{V_0t^\alpha }}{{{\mathrm{exp}}}}\left( { - \frac{{Vt_0}}{{V_0t^\alpha }}} \right).$$

We note that this is compatible with the self-similar dynamical scaling form proposed by other studies^43,45^, who proposed that the distribution *f*(*s*, *t*) of cluster size *s* at time *t* can universally be described as $f\left( {s,t} \right) = t^\xi \psi \left( {\frac{s}{{t^z}}} \right)$ for some function *ψ* (refs. ^43,45^).

Here we have shown that both native nuclear speckles and synthetic condensates form via a quench-then-coalesce mechanism. The universality of this distribution, which fundamentally arises from coarsening dynamics, suggests that the cell may be able to dynamically control the droplet size by manipulating the number of droplets formed and the total amount of available droplet material. Indeed, several native organelles within the nucleus, including nuclear speckles^9^ but also nucleoli^24^, Cajal bodies^24^, histone locus bodies, paraspeckles and promyelocytic leukaemia (PML) bodies, disassemble or scatter into the cytoplasm during mitosis^19^, probably due to a combination of dilution and post-translational modifications^20,46^, and then nucleate and regrow in the resulting daughter nuclei. Our results show that a relatively narrow, exponentially decaying size distribution will be attained for simple regrowth driven by coalescence. Furthermore, the growth of the mean condensate size will be slowed if the motion of condensates is subdiffusive, as is common in cells^21,47^, which may allow for a quasi-static mean condensate size that only changes modestly over the biologically relevant timescales such as the cell cycle. Finally, although condensate nucleation often occurs at specific sites, for example, at defined genomic loci, these loci themselves also undergo subdiffusive Brownian motion^34,48^. In general, we propose that the maintenance of an exponential size distribution of condensates by slow coalescence may be conveniently implemented given the natural dynamics of the cell cycle, irrespective of the molecular details of the condensate.

By contrast, with the simple exponential distribution with a single mean size, power-law distributions are broad and scale free, indicative, in this context, of a preferential attachment effect, where coalescence events are biased towards larger, existing condensates. We demonstrated two possible causes of this effect. In the case of the neurodegeneration-associated mutant Htt-polyQ species, we observed that ongoing nucleation effectively gives older condensates more time to grow, resulting in preferential attachment and a power-law distribution. There is not yet a consensus on whether small oligomers or larger Htt-polyQ aggregates are to blame for accelerated disease progression or are a compensatory mechanism to slow cytotoxicity^49,50^. Regardless, the slow, steady production of aggregate generates a broad power-law distribution. We also suggest that preferential attachment is possible when diffusion is highly non-Stokesian. In general, the observation of power laws in condensates may be attributed to either or both these mechanisms or to others that have yet to be observed in cells, such as Ostwald ripening, which would effectively result in the merger of small and large condensates. However, the measurement of exponentials in nuclear bodies is consistent with the observation that Ostwald ripening is not a major contributing factor in the nucleus^21,22^.

The distribution of condensate sizes is also relevant to other cellular pathologies, for example, in nucleolar size changes in diseases such as progeria and cancer^10–12^. Here, too, it is unclear whether the observed increase in condensate size in these contexts is causative or purely correlative. This size increase may be indicative of altered coalescence or production dynamics, which might also be responsible for dysfunction. For instance, an exponential form with a larger mean might be indicative of an increased subdiffusive exponent, for example, due to reduced nuclear elasticity that previously has been associated with cancer, whereas a broadened distribution might be indicative of a preferential attachment process. The mechanical environment of the nucleus could have still more complex effects on the size distribution: local heterogeneities in the chromatin stiffness could lead to preferential nucleation in specific locations^22,23^, locally altering the size dependence of merger probabilities and hence changing the form of distribution. Moreover, the local pore size of chromatin could impose a size cutoff on the distribution.

Moreover, other factors, such as non-equilibrium activity and condensate-dependent reaction rates^51^, have been described as potential regulators of condensate size, probably inhibiting coalescence and growth. Some active processes could be captured by the framework described here: if active processes facilitate the coalescence of larger condensates, for example, by speeding their diffusion or making them superdiffusive, and/or suppress the coalescence of smaller condensates, the distribution would become wider and more power-law like, whereas if the opposite were true, the distribution would become more exponential. For example, nucleoli are commonly observed to exhibit an unexpected power-law distribution in size, which probably results not only from coalescence dynamics but also from the strong influence of regulated steady-state production of ribosomal RNA. Similarly, droplets formed from associative polymers, for example, as considered in magic number^52^ or sticker-and-spacer^53^ models, may also reach metastable sizes as interaction surfaces become saturated, preventing growth by collisions^54^. Examining such size distributions under perturbations that modulate biological activity represents an exciting next frontier, which will yield newer insights into the coupling of classical materials’ coarsening dynamics and associated biological function and dysfunction.

## Methods

### Plasmids

Here eGFP-polyQ74 was an adaptation of a gift from David Rubinsztein (Addgene plasmid #40262) with the insertion of N-terminal residues MATLEK as a part of the N17 domain and C-terminal residues PQAQPLLPQPQPPPPPPPPPPGPAVAEEPLHRP that comprise the proline-rich domain containing the C38 domain using primers as part of the In-Fusion cloning protocol (Takara). Also, eGFP-polyQ31 was adapted from eGFP-polyQ74 through the variability of Q-length PCR products amplified using CloneAmp HiFi PCR Premix (Takara). FM5-NPM1-mCh was generated as described elsewhere^55^.

To generate an sgRNA plasmid to target SRRM2, a guide RNA duplex was designed to insert EYFP at the beginning of the endogenous human SRRM2 coding sequence and then cloned downstream of the U6 promoter in the PX458 vector (Addgene Plasmid #48138; gift from Feng Zhang lab). The annealed oligonucleotides used to generate the final ‘pU6-SRRM2gRNA-CMV-FLAG-NLS-SpCas9-2A-mGFP’ plasmid were as follows:

**Table:** 

SRRM2 sgRNA Forward	5’ CACCGGGCCATGTACAACGGGATC 3’
SRRM2 sgRNA Reverse	5’ AAACGATCCCGTTGTACATGGCCC 3’

Separately, a gene fragment containing upstream and downstream SRRM2 homology arms, flanking the full-length EYFP coding sequence, was synthesized (IDT) and then cloned into the pUC19 vector, first digested with HindIII and BamHI. The following oligonucleotides were used to create the final ‘pUC19-SRRM2 NonCode Homology-EYFP-SRRM2 Exon1 Homology’ plasmid:

**Table:** 

SRRM2 homology forward	5’ TTGATGATAAGCTTcctttcttcaccactgagctccttcaaggg 3’
SRRM2 homology reverse	5’ TTGATGATGGATCCccatagcctgcatgtccactcccagacgatgg 3’

Sanger sequencing (GENEWIZ) confirmed gene insertions in full for all the constructs.

### Cell culture and cell-line generation

U2OS (a kind gift from Mark Groudine lab, Fred Hutchinson Cancer Research Center), HeLa CCL-2 (ATCC), HEK-D (as shown below) and Lenti-X 293T (Takara) cells were cultured in a growth medium consisting of Dulbecco’s modified Eagle’s medium (GIBCO), 10% foetal bovine serum (Atlanta Biologicals) and 10 U ml^–1^ penicillin–streptomycin (GIBCO), and incubated at 37 °C and 5% CO_2_ in a humidified incubator.

#### iPSC culture

Here iPSCs were obtained from the Allen Institute for Cell Science at Coriell Institute. The iPSC line AICS-0094-024 (Mono-Allelic mEGFP-tagged SON WTC iPSC line) was used for our experiments. The colonies were expanded and maintained on Matrigel (Corning) in mTeSR Plus medium (Stem Cell Technologies). The cells were plated at 3,000–10,000 cells per square centimetre to obtain ~75% confluency every 5–7 days. The cells were passaged using ReLeSR (Stem Cell Technologies) and split at a 1:10–1:50 ratio. The mTeSR plus medium was supplemented with ROCK inhibitor Y-27632 (Selleck Chemicals) for a maximum of 24 h after cryopreservation or passaging. Then, iPSCs were cryopreserved in mTeSR Plus medium supplemented with 30% KnockOut Serum Replacement (Gibco Life Technologies) and 10% dimethyl sulfoxide.

#### CRISPR-Cas9-based generation of HEK293 cells expressing eYFP-tagged SRRM2

Single HEK293 cells (kind gift from Marc Diamond lab, UT Southwestern) were isolated by fluorescence-activated cell sorting. A clonal line termed HEK-D was selected for its relatively flat morphology and preferable imaging characteristics. The cell-line background was validated by short tandem repeat profiling (America Type Culture collection).

To generate the EYFP-tagged SRRM2 clonal line, HEK-D cells were plated in a 24-well dish to achieve 70% confluency the next day. Lipofectamine 3000 (Thermo Fisher) was used to transfect the two plasmids at a one-to-one ratio, according to the manufacturer’s recommendation. The cells were amplified by successive passages to six-well dishes and then to 10 cm dishes. On approximately day 10 following transfection, the cells were trypsinized, pelleted and then resuspended in the flow cytometry buffer (Dulbecco’s phosphate-buffered saline with 10% foetal bovine serum). Single eYFP-positive cells were sorted by fluorescence-activated cell sorting (Flow Cytometry Resource Facility, Princeton Department of Molecular Biology) into separate wells of 96-well plates. The resulting colonies with the expected localization of eYFP signal to nuclear speckles (but not cytoplasm) were amplified, and a single clone with a relatively high fluorescence intensity was selected (eYFP-SRRM2 48 HEK D).

To validate, the cells were passaged into single wells of a 96-well plate containing either 200 µl of pCRISPRv2-SRRM2 gRNA lentivirus (pooled, six gRNAs designed according to the published recommendations^56^) or 200 µl of pCRISPRv2-NonTarget gRNA lentivirus. After 72 h, the confluent cells were washed, trypsinized and passaged at a 1:8 dilution factor. After 96 h, the confluent cells were plated onto a fibronectin-coated, glass-bottom, 96-well plate (MatTek), and eYFP fluorescence was compared between the experimental (SRRM2 KO) and control (NonTarget) conditions, with confocal microscopy performed at seven days post-lentivirus transduction. The eYFP fluorescence was completely abrogated in the SRRM2 knockout (>90% of cells) condition, suggesting that eYFP was only integrated at the endogenous SRRM2 loci.

Next, whole-genome sequencing assessed the number of tagged copies in the hypotriploid HEK293 clone featuring three copies of chromosome 16, on which the SRRM2 gene is located. Two of the three copies contained the exogenous EYFP sequence between chr16:2,756,364 and chr16:2,756,365, corresponding to immediately before endogenous SRRM2’s start codon and after the expected upstream non-coding sequence. CRISPR-Cas9-mediated genome editing left single-nucleotide polymorphism encoding a synonymous codon (Gly6Gly) in SRRM2-tagged loci, otherwise unaltered from the parental sequence. The cells were then cultured and imaged as described elsewhere.

### Lentiviral transduction

For Corelet, Htt-polyQ and NPM1 overexpression, lentivirus was produced by transfecting the transfer plasmids pCMV-dR8.91 and pMD2.G (mass ratio, 9:8:1) into Lenti-X cells grown to approximately 80% confluency in six-well plates using FuGENE HD Transfection Reagent (Promega) as per manufacturer’s protocol. A total of 3 μg plasmid and 9 μl transfection reagent were delivered into each well. After 60–72 h, supernatant containing the viral particles was harvested and filtered with a 0.45 μm filter (Pall Life Sciences). The supernatant was immediately used for transduction or aliquoted and stored at −80 °C. The cells were seeded at 10% confluency in 96-well plates and 20–200 μl filtered viral supernatant was added to the cells. Media containing the virus was replaced with fresh growth medium 24 h post-infection. The infected cells were imaged no earlier than 72 h after infection. For Htt-polyQ HeLa lines, the cells were transferred to a glass 96-well imaging plate coated with fibronectin 2 days following transduction and imaged 4–6 days post-transduction.

### Microscopy

Images of Corelets, nucleoli and nuclear speckles were taken with a spinning-disc (Yokogawa CSU-X1) confocal microscope with a ×100 oil-immersion Apo total internal reflection fluorescence objective (numerical aperture, 1.49) and an Andor DU-897 electron-multiplying charge-coupled device camera on a Nikon Eclipse Ti body. A 488 nm laser was used for imaging GFP and global activation, and a 561 nm laser was used for imaging mCherry. The imaging chamber was maintained at 37 °C and 5% CO_2_ (Okolab) with a 96-well plate adaptor. The *Z* stacks were taken every 300 nm for 15 μm using an ASI MS-2000 stage controller. For drug treatments, DRB (Sigma) or ActD (Sigma) were dissolved in dimethyl sulfoxide at 50 and 2 mg ml^–1^ stock concentrations, respectively, which were diluted in complete Dulbecco’s modified Eagle’s medium. The cells were treated by replacing the media and incubating for 4 h before imaging.

Images of Htt-polyQ were taken on a Nikon A1 laser scanning confocal microscope using a ×60 oil-immersion lens with a numerical aperture of 1.4. A 488 nm laser was used for imaging GFP. The imaging chamber was maintained at 37 °C and 5% CO_2_ (Okolab) with a 96-well plate adaptor. The *Z* stacks were taken every 420 nm for 15 μm using an ASI MS-2000 stage controller.

### Image analysis

All the images were analysed in Fiji (ImageJ 1.52p)^57^ and MATLAB 2019b (Mathworks).

#### Corelets

Data were reanalysed from previous work^21^. Individual nuclei were cropped by hand and saved as .tif files, which were analysed in MATLAB. The droplets were segmented in the GFP channel by using Otsu’s algorithm to identify the nucleus in the first frame; an intensity threshold was defined as two standard deviations above the mean of the initial nuclear GFP intensity. This threshold was applied to all the subsequent frames to identify droplets; regions of 4 pixels and smaller were discarded. Then, regionprops was used to identify individual domains and calculate their area. The volumes were calculated by assuming perfect sphericity, based on previous work^21^; distributions were then calculated for each nucleus.

#### Nucleoli and nuclear speckles

Individual nuclei were cropped by hand and saved as .tif files, which were analysed in MATLAB. Maximum projects were generated and used to calculate a threshold for each nucleus and channel, which was set to the mean of the image plus two standard deviations. This threshold was applied in three dimensions to each *Z* stack; regionprops3 was used to calculate the volume of each condensate. Condensates below 20 voxels in size were discarded. CDFs were calculated for each nucleus, rescaled by mean volume and averaged over the nuclei. The error was propagated as s.e.m.

#### PolyQ aggregates

Individual cells were cropped by hand and saved as .tif files, which were analysed in MATLAB. A frame of the maximum mean cytoplasmic brightness was selected to calculate a threshold for the cytoplasm, which was set to the mean of the image plus 2.5 standard deviations. This threshold was applied in three dimensions to each *Z* stack; regionprops3 was used to calculate the volume of each aggregate. Aggregates below 16 voxels in size were discarded. CDFs were calculated for each nucleus, rescaled by mean volume and averaged over the cellular cytoplasms. The error was propagated as s.e.m.

### Simulations

Simulations were performed in MATLAB on the Della cluster (Princeton Research Computing). First, an initial configuration of spheres was generated in three dimensions with periodic boundary conditions, with initial sphere volumes drawn from a uniform distribution ranging from 1 to 2 a.u. The box size was set based on the volume fraction (generally set at 5%). Overlapping spheres were then merged. Sphere merger was implemented by randomly selecting a pair of overlapping spheres and replacing them with one new sphere centred at the centre of mass of the pair. The size of the new sphere was determined by volume conservation of the original pair. This was iterated until no pairs of overlapping spheres remained (for ‘vanishing’ simulations employed as controls (Extended Data Figs. 3 and 4), all the spheres were set to a volume of 1 a.u. and maintained at a volume of 1 a.u. after merger, resulting in some spheres effectively ‘vanishing’). Then, for each sphere, a fractional Brownian motion trajectory with appropriate *α* was synthesized (using the wfbm function from MATLAB’s wavelet toolbox, which utilizes the algorithm detailed elsewhere^32^) for the appropriate number of timesteps (generally 10^5^). At each timepoint, each sphere proceeded one step along the synthesized trajectory, scaled such that *D* ≈ 1/*R*^*γ*^. Spheres were merged as previously described. The merged spheres inherited the predetermined trajectory from one of their parent spheres (chosen arbitrarily), moving with the appropriate step size. This was repeated for the duration of the synthesized trajectories.

For injection simulations, the simulation box was initially empty; *J* spheres were initialized per timestep in randomly generated locations (that is, if *J* = 0.25, a sphere was initialized at every fourth timestep, whereas for *J* = 4.00, four spheres were initialized per timestep). Then, mergers and sphere diffusion proceeded as previously described before the next injection.

Size distributions were recorded for 20 replicates of each simulation condition, binned and averaged before calculating the CCDFs. To calculate the collision rate constant, 960 (20 in the case of the ‘vanishing’ control simulations) simulation replicates were performed; for each merger event, the sphere sizes of the merging pair and number of spheres of those sizes were recorded. Each merger event occurring between spheres of volume *V*_1_ and *V*_2_ was weighted by a factor of 1/(*f*(*V*_1_)*f*(*V*_2_)), where *f* gives the number density of spheres of a particular size. Summing over weights of all the merger events in each simulation replicate and then averaging over replicates gave an estimate of the coagulation kernel value after normalization by system size and simulation duration (Supplementary Note).

## Online content

Any methods, additional references, Nature Portfolio reporting summaries, source data, extended data, supplementary information, acknowledgements, peer review information; details of author contributions and competing interests; and statements of data and code availability are available at 10.1038/s41567-022-01917-0.

## Supplementary information

Supplementary Information Supplementary Note.

## Data Availability

Data, reagents and analysis code associated with this work are available from the corresponding authors upon reasonable request.

## Code availability

Simulation code associated with this work is publicly available via GitHub at https://github.com/dswlee0519/Condensate_Size_Distribution_Simulations.
