# Supplementary material for: Size distributions of intracellular condensates reflect competition between coalescence and nucleation
Source: Nat Phys. 2023 Feb 2;19(4):586–96. doi: 10.1038/s41567-022-01917-0 (PMC10104779; doi:10.1038/s41567-022-01917-0)
Supplement: Supplementary file 1 — Supplementary Note. [file 41567_2022_1917_MOESM1_ESM.pdf]

nature physics

Article https://doi.org/10.1038/s41567-022-01917-0

Size distributions of intracellular condensates reflect competition between coalescence and nucleation

In the format provided by the authors and unedited

Supplementary Information

Supplementary Notes

Exponential distribution with size cutoff

An exponential distribution is given by:

$$f(x) = \frac{1}{\lambda} \exp\left(\frac{-x}{\lambda}\right).$$

We consider an exponential distribution with a size cutoff at size x_0

$$f(x) = \begin{cases} 0 \text{ if } x < x_0 & \text{if } x < x_0 \\ \frac{\aleph}{\lambda} \exp\left(\frac{-x}{\lambda}\right) & \text{if } x > x_0 \end{cases}$$

for some normalization factor $\aleph$. To calculate $\aleph$, we realize that $\int_0^\infty f(x) = 1$, and therefore $\int_{x_0}^\infty \aleph(\frac{1}{\lambda} \exp \frac{-x}{\lambda})dx = 1$, giving $\aleph = \exp\left(\frac{x_0}{\lambda}\right)$.

Finally, to calculate the average,

$$\langle x \rangle = \int_{x_0}^\infty \frac{1}{\lambda} \exp\left(\frac{-(x-x_0)}{\lambda}\right) x dx = \lambda + x_0,$$

and thus the mean is merely shifted, justifying a shift in the mean-variance plot corresponding to a minimum detection threshold (which can be applied computationally at a size scale greater than the diffraction limit to ensure proper sampling).

Higher central moments of the distribution are given in general by $\mu_n = \langle x - \langle x \rangle^n \rangle$, which should then be unchanged.

Derivation of monomer kinetics

The time evolution of monomer concentration is given by

$$\frac{\partial n}{\partial t}(V_0, t) = j - 2K(V_0, V_0)n(V_0, t)n(V_0, t) - \int_0^\infty K(V_0, V)n(V_0, t)n(V, t)\Theta(V - V_0)dV,$$

where V_0 is monomer size, j is the total rate of monomer injection divided by system volume, and Θ is the Heaviside step function; the latter two terms describe the loss of monomeric spheres to diffusive merger processes (no monomers are gained by merger because there is no sphere fission and spheres are a minimum volume of V_0 when injected). For the merger kernel, we have

$$K(V_1, V_2) = 4\pi(D_1 + D_2)(R_1 + R_2).$$

We can rewrite this as $K(V_1, V_2) = K(\alpha)\left(\frac{1}{R_1} + \frac{1}{R_2}\right)(R_1 + R_2) = K_\alpha \frac{(R_1+R_2)^2}{R_1 R_2}$ where $K(\alpha)$ is a prefactor containing the diffusion coefficient. For monomer-monomer collisions, $R_1 = R_2$. Therefore,

$$\frac{\partial n}{\partial t}(V_0, t) = j - 4K(\alpha)n(V_0, t)^2 - K(\alpha)n(V_0, t)\int_{V_0}^\infty \frac{(R_0 + R)^2}{R_0 R} n(V, t)dV.$$

At very early times, clusters will primarily be small so the third term is negligible. Similarly, at early times, the predominant population, of clusters is monomeric, so $n(V_0, t) \approx \rho$, and we get:

$$\frac{\partial n}{\partial t}(V_0, t) = j - 4K(\alpha)\rho^2.$$

Subsuming the numerical factor into the definition of $K(\alpha)$, we then define $\chi \equiv \frac{\tau_{\text{inject}}}{\tau_{\text{merge}}} = \frac{\rho^2 K(\alpha)}{j}$ as in the main text; thus

$$\frac{\partial n}{\partial t}(V_0, t) = j(1 - \chi).$$

The injection rate j is strictly positive, so for $\chi < 1$ the concentration of monomeric spheres grows since they are injected faster than merger. However, for $\chi > 1$ the concentration of monomeric spheres becomes depleted since merger is faster than injection. This results in an increasing mean sphere size which continues to grow.

This can be further demonstrated by considering the limit $R_0 \ll R_1$. In this case, the monomer-monomer collision term is negligible and $\frac{(R_1+R_0)^2}{R_1 R_0} \approx \frac{R_1}{R_0}$, and so:

$$\frac{\partial n}{\partial t}(V_0, t) = j - K_\alpha n(V_0, t) \int_0^\infty \frac{R_1}{R_0} n(V, t) dV = j - K_\alpha n(V_0, t) \frac{\langle R(t) \rangle}{R_0}.$$

This suggests that in the regime of $\chi < 1$, the mean will be driven higher with time as $\langle R(t) \rangle$ grows to be much larger than R_0, resulting in a preferential attachment effect as small spheres are lost increasingly quickly.

Kernel Normalization

The number of collisions M between species of volumes V_1 and V_2 is given by

$$\frac{M(V_1, V_2)}{V_{sys}} = \sum_{V_1, V_2} \int_t K(V_1, V_2) f(V_1, t) f(V_2, t) dt.$$

Then $I(V_1, V_2)$ empirically counts collisions between populations of volumes V_1 and V_2, so

$$I(V_1, V_2) = K(V_1, V_2) V_{\text{sys}} f(V_1, t) f(V_2, t) \int dt,$$

and thus the number of collisions counted over a period of time T in a system of volume size V_{sys} is given by:

$$K(V_1, V_2) = \frac{I(V_1, V_2)}{f(V_1, t) f(V_2, t) V_{\text{sys}} T}.$$
